# Cutaneous adverse reactions in B-RAF positive metastatic melanoma following sequential treatment with B-RAF/MEK inhibitors and immune checkpoint blockade or vice versa. A single-institutional case-series

**DOI:** 10.1186/s40425-018-0475-y

**Published:** 2019-01-08

**Authors:** Abdul Rafeh Naqash, Danielle M. File, Carolyn M. Ziemer, Young E. Whang, Paula Landman, Paul B. Googe, Frances A. Collichio

**Affiliations:** 1 Department of Medicine, Division of Hematology/Oncology, Vidant Cancer Center at East Carolina University, 600 Moye Blvd, Greenville, NC 27834 USA; 2 Department of Medicine, Division of Hematology/Oncology, University of North Carolina, 170 Manning Drive, Chapel Hill, NC 27599 USA; 3 Department of Dermatology, University of North Carolina, Chapel Hill, NC 27516 USA; 4 Department of Medicine, Division of Hematology/Oncology, Lineberger Comprehensive Cancer Center, University of North Carolina, 170 Manning Drive, Chapel Hill, NC 27599 USA; 5 UNC Health Care, The University of North Carolina, 170 Manning Drive, Chapel Hill, NC 27599 USA; 6 Department of Dermatology, Pathology and Laboratory Medicine, University of North Carolina School of Medicine, 250 Bell Tower Drive CB#7287, Chapel Hill, NC 27599 USA

**Keywords:** Cutaneous adverse events, Metastatic melanoma, B-RAF inhibitors, Immune checkpoint blockade, Vemurafenib/cobimetinib, DRESS syndrome

## Abstract

**Background:**

With the advent of immune-checkpoint inhibitors and targeted treatments (TT), there have been unprecedented response rates and survival in advanced melanoma, but the optimal sequencing of these two treatments modalities is unknown. Combining or sequencing these agents could potentially result in unique toxicities. Cutaneous adverse events (CAE) after sequential exposure to these agents represents one toxicity that needs further description.

**Methods:**

After retrospectively reviewing charts of patients from 2015 to 2018, we identified six patients who experienced CAEs after recent exposure to sequential immunotherapy and TT or vice versa for the treatment for metastatic melanoma at the University of North Carolina, Chapel Hill. Skin biopsies were available in five patients.

**Results:**

Five patients received TT after immunotherapy, and one patient received immunotherapy after TT. TT consisted of vemurafenib/cobimetinib (V/C) in five patients with four patients starting V/C immediately before manifesting with a CAE. In patients receiving V/C after immunotherapy, the median time from beginning V/C to development of CAE was 14.5 days. The clinical presentation of diffuse morbilliform rash, fevers, hypotension, and end-organ damage raised concern for Drug Reaction with Eosinophilia and Systemic Symptoms (DRESS) syndrome. Histopathological features of lympho-eosinophilic infiltrate were supportive of a drug eruption. Immunotherapy or TT were re-initiated in five patients within 1–8 weeks after resolution of the index CAE. This resulted in two patients re-experiencing the CAE. Both of these patients were off prednisone at the time of therapy re-initiation, whereas none of the patients who were restarted on targeted therapy with a steroid overlap had a rash recurrence.

**Conclusions:**

Sequential treatment using immunotherapy and TT, especially the sequence of V/C after immunotherapy appears to be the most common trigger for CAE with a median time to onset of approximately 2 weeks. Although the clinical presentation of these CAEs can be dramatic, they respond well to prednisone therapy. This unique presentation suggests that it may be reasonably safe to re-challenge certain patients with a steroid overlap after rash resolution.

## Introduction

With the advent of immune-checkpoint inhibitors and novel targeted therapies (TT), the treatment landscape for melanoma has witnessed a paradigm shift with unprecedented survival and response rates both in the adjuvant and the metastatic settings [1]. This, in turn, has prompted a revolution that has gradually spread across certain other tumor types, resulting in remarkably improved outcomes. Around 40–50% of cutaneous melanomas harbor mutations in the B-RAF gene with V600E accounting for 80–90% in this category. One of the standard approaches for B-RAF V600E mutated melanomas involves using B-RAF inhibitors in conjunction with MEK inhibitors [2]. Immune-checkpoint blockade (ICB) involving the use of monoclonal antibodies to modulate the immune checkpoints such as programmed cell death-1 (PD-1) and cytotoxic T-lymphocyte antigen-4 (CTLA-4) is the other rapidly evolving approach that has demonstrated strong potency and durable efficacy in the frontline treatment of advanced melanomas including B-RAF mutated melanomas [1, 3].

In addition to various clinical factors, determining the primary and the salvage agent of choice to treat advanced-stage B-RAF V600E melanomas can be guided by the differential activities of ICB and TT. For example, TT have a rapid onset of action while ICB produces sustained responses providing an overall survival “tail” [1, 4]. Also, in response to disease progression, sequencing ICB and TT in B-RAF mutated melanoma is a reasonable approach frequently practiced in the real-life setting. The clinical benefit of a sequencing approach is supported by compelling pre-clinical data demonstrating the immune modulatory effects of TT that may serve to augment an endogenous anti-tumor response and thus achieve synergy with ICB [5, 6]. Presently, however, the paucity in efficacy and toxicity data from randomized studies tends to be a barrier in implementing these sequential or combination strategies into regular clinical practice.

In our case series, we focus on patients treated with ICB followed by TT, or vice versa, who experienced cutaneous toxicities with unique histological patterns, a feature that has not been well described in the literature. We also attempt to understand certain common denominators that could have contributed to these presentations. Furthermore, we explore possible mechanisms of toxicity and diagnostic pitfalls that could help guide management in such scenarios.

## Materials and methods

After institutional review board approval, we retrospectively reviewed charts from March 2015 to September 2018 to identify patients experiencing cutaneous adverse events (CAE) on receiving sequential ICB and TT or vice versa for the treatment of metastatic melanoma at the University of North Carolina, Chapel Hill. We identified five patients who received TT after ICB and one patient who received ICB after TT. Skin biopsies were available in five patients for better characterization of the cutaneous reactions. We also collected relevant data on laboratory parameters, clinical course, and management.

## Results

**Fig. 1:**
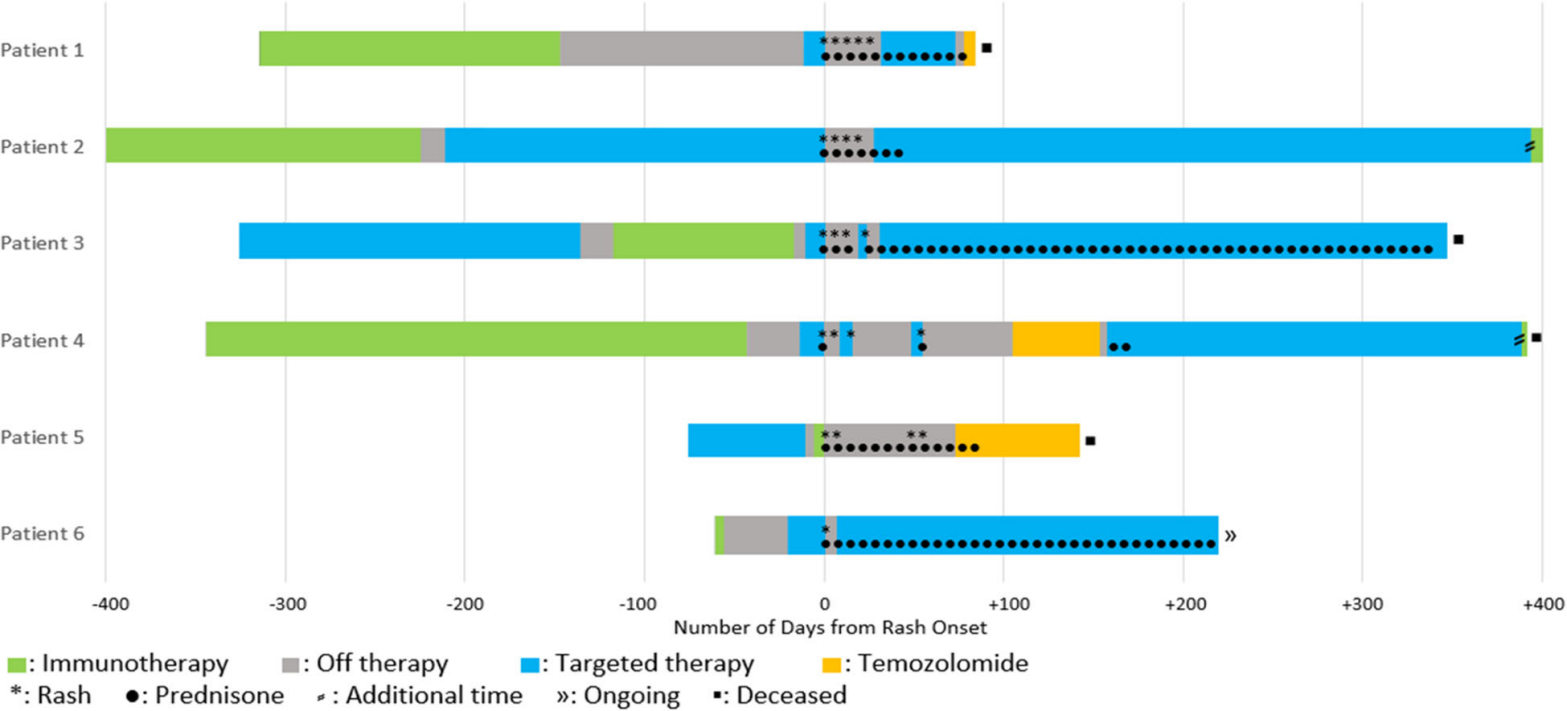
Therapy timelines for patients in relation to rash onset showing sequencing of ICB and TT

**Fig. 2:**
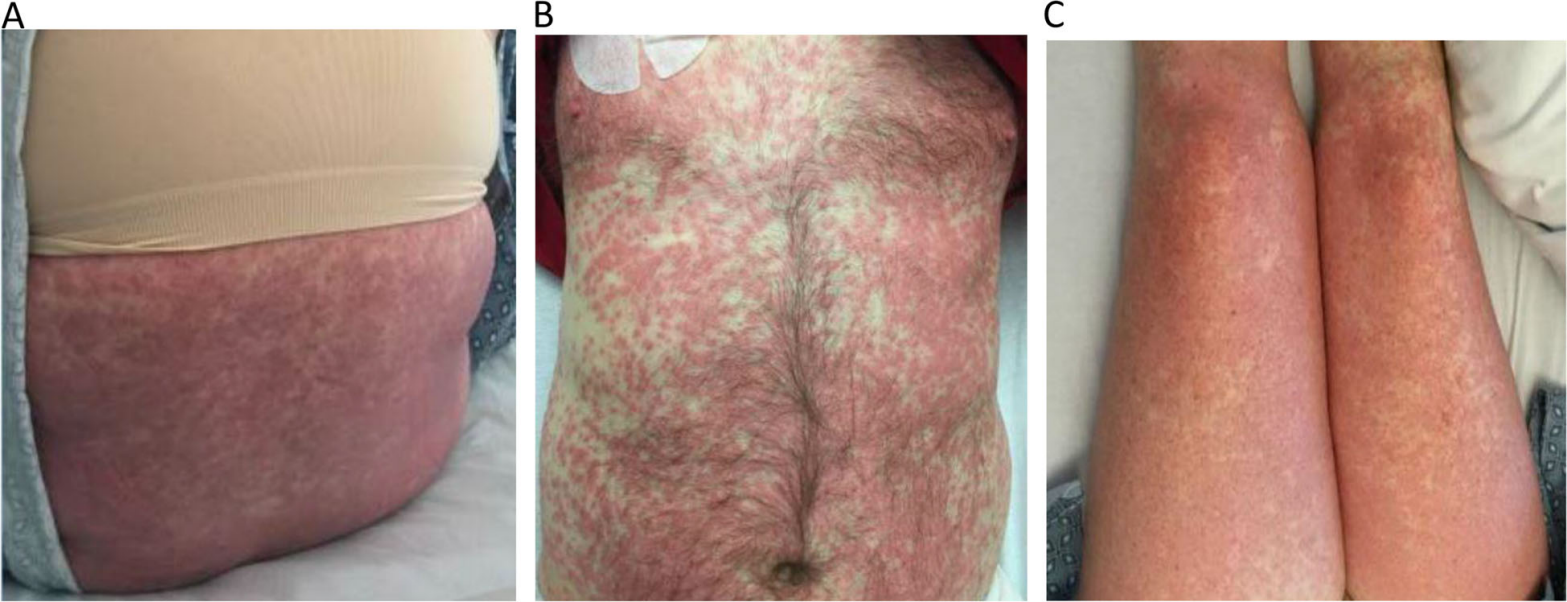
Diffuse morbilliform eruption involving trunk (**a**, **b**) and extremities (**c**)

**Fig. 3:**
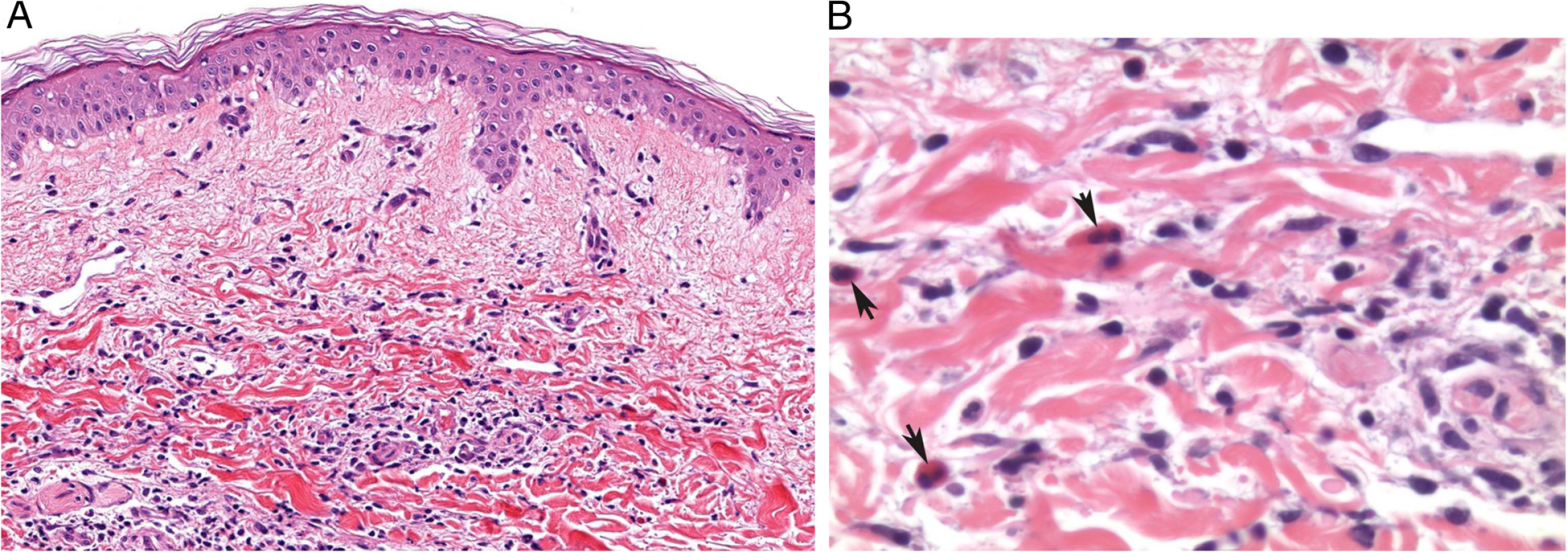
Skin biopsy of rash with histology. **a** Skin biopsy shows slight basal layer vacuolization, dermal edema and a superficial dermal perivascular lymphocyte and eosinophil infiltrate. No necrosis is present. (H&E, 200X). **b** Eosinophils (arrows) are present with lymphocytes around the superficial dermal capillaries fibrinoid necrosis of capillary walls, a sign of vasculitis, is not present. (H&E, 400X)

(Table 1) All our patients were of Caucasian ethnicity with a median age of 58 years when diagnosed with CAE. Five of the six patients were female. The sequence of treatment timeline and CAE are depicted in Fig. 1. A total of five cases (patients 1–4 and patient 6) received ICB before TT as the two most recent treatment modalities before experience a CAE. Only Patient 5 received ICB after TT. In the five patients who received ICB before TT, the median time from discontinuation of the most recent ICB to CAE was 56 days (24–228 days). In four patients (patients 1–4) nivolumab was given for ICB before TT. Pembrolizumab was utilized for ICB in two patients, with patient-5 receiving it after TT and patient-6 receiving it before TT. ICB was primarily used as a single agent with the only exception being patient-1 where one dose of ipilimumab had been given with nivolumab followed by nivolumab alone. Before switching to TT, the median number of ICB doses for the five patients receiving TT after ICB was 12 (1–22 doses). In our cohort, TT consisted of vemurafenib/cobimetinib (V/C) in five patients (patients-1 and 3–6) with four patients (patients 1, 3, 4 and 6) receiving V/C immediately before manifesting with a CAE. In patients that specifically received TT in the form of V/C after ICB, the median time from starting V/C to development of CAE was 14.5 days (12–21 days). Patient 2 was on dabrafenib/trametinib (D/T) for a considerable period (214 days) before developing the CAE. The timeline for the rash in patient-2 coincided with starting amoxicillin/clavulanic acid for a pneumonia. Patient-5 developed the CAE immediately after one dose of pembrolizumab. Patient-3 had been previously treated with D/T before switching to ICB but tolerated both these agents for a considerable time without any toxicity. However, when switched from ICB to V/C, he manifested with the rash immediately within 16 days from the switch. All four patients who presented with signs and symptoms consistent with systemic inflammatory response syndrome (SIRS) including high-grade fevers, hypotension, and end-organ damage required hospitalization (patients 1, 2, 3 and 5). The values for C-reactive protein were available in three patients with all values measuring > 150 mg/L (reference range < 10.0 mg/L). Oral mucositis was an accompanying feature in two of the six patients (patients 1 and 2). Most of the patients had grade-4 CAE based on the Common Terminology Criteria for Adverse Events (CTCAE 4.03) system (Table 1). The CAE was mainly characterized by a diffuse erythematous morbilliform eruption involving ≥30% body surface area (Fig. 2) with histopathology primarily comprising of a lympho-eosinophilic infiltrate (Fig. 3b). Patient 2 was the only exception where sub-epidermal vesicle formation suggestive of bullous pemphigoid was seen. As part of a comprehensive assessment, patient-1 had serum cytokines measured, and notably, the level for the interleukin-2 receptor (IL-2R) was seen to be > 20 times the upper limit (Table 1).

**Table 1:** Therapy and Rash Characteristics

Patient	Demographics at Time of Rash Onset	Therapy Sequence [(Days to Rash Onset (RO)]	Clinical Course	Lab Abnormalities	CTCAE Rash Grade (G), Characterization Location Initial Diagnosis	Histopathology	Confounding Variables	Current Survival Status
1	54 year old Caucasian female	I/N ×1 - > N (Day − 315 to − 148) V/C (Day −12 to RO)	Hospitalized High grade fever Hypotension Oral mucositis	Hyponatremia Hypokalemia Elevated lactate (2.5XULN) Lymphocytopenia Elevated sIL-2R (20 X ULN) CRP 163	G-4, Diffuse morbilliform eruption of trunk and extremities DRESS vs SJS vs drug reaction	Papillary dermal edema, slight basal layer vacuolization, superficial dermal perivascular lymphocytic infiltration and eosinophils	Started allopurinol 14 days prior to rash onset	Deceased
2	60 year old Caucasian female	N (Day − 408 to −228) D/T (Day −214 to RO)	ICU High grade fever Hypotension AMS Lip swelling Difficulty swallowing	Transaminitis	G-4, Diffuse morbilliform eruption of trunk and extremities Drug reaction vs sepsis vs DRESS	Subepidermal vesicle formation with eosinophils; positive direct immunofluorescence with linear IgG and IgA at the basement membrane zone suggestive of bullous pemphigoid or linear IgA bullous dermatosis	On course of amoxicillin-clavulanic acid at rash onset	Alive
3	54 year old Caucasian male	D/T (Day − 331 to − 143) N (Day − 122 to − 24) V/C (Day − 16 to RO)	Hospitalized High grade fever	Hyponatremia Elevated creatinine Lymphocytopenia	G-4, Diffuse targetoid lesions involving trunk, extremities and face SJS vs drug eruption (including DRESS) vs viral exanthem	Papillary dermal edema, slight basal layer vacuolization, superficial dermal perivascular lymphocytic infiltration and eosinophils	Symptoms of upper respiratory tract infection present immediately prior to rash onset	Deceased
4	58 year old Caucasian female	N (Day − 344 to −43) V/C (Day −13 to RO)	Outpatient management	None	G-4 Diffuse morbilliform eruption of trunk and extremities Drug reaction	No biopsy performed	Started with full doses of V/C when had been instructed to start at a reduced dose of each	Deceased
5	59 year old Caucasian female	V/C (Day −75 to −11) P (Day −6 to RO)	ICU High grade fever Hypotension Tachycardia	Elevated creatinine Transaminitis Hyperbilirubinemia CRP 200	G-4 Diffuse morbilliform eruption of trunk and extremities Sepsis vs DRESS vs pembrolizumab induced reaction	Papillary dermal edema, slight basal layer vacuolization, superficial dermal perivascular lymphocytic infiltration and eosinophils; few plasma cells, neutrophils, and a rare focus of parakeratosis	Concern for sepsis from cellulitis (re-fevered after antibiotics narrowed)	Deceased
6	58 year old Caucasian female	P (Day −56) V/C (Day −21 to RO)	Outpatient management	Transaminitis Lymphocytopenia CRP 159	G-2 Discrete scattered erythematous edematous plaques on extremities Drug reaction vs Sweets Syndrome vs early SJS	Papillary dermal edema, slight basal layer vacuolization, superficial dermal perivascular lymphocytic infiltration and eosinophils	No apparent confounding variables	Alive

All days are calculated from rash occurrence (RO) being day 0, *I* Ipilimumab, *N* Nivolumab, *P* Pembrolizumab, *V* Vemurafenib, *C* Cobimetinib, *D* Dabrafenib, *T* Trametinib, *sIL-2R* Soluble interleukin-2 receptor, *ULN* Upper limit of normal, High grade fever= > 102, *AMS* Altered mental status, *CRP* C-reactive protein (reference range < 10.0 mg/L), *SJS* Stevens Johnson syndrome, *DRESS* Drug Reaction with Eosinophilia and Systemic Symptoms, *CTCAE* Common Terminology Criteria for Adverse Events (CTCAE 4.03)

**Table 2:** Rash Treatment and Outcome

Patient	Supportive Measures	Treatment	Total Steroid Duration	Duration of Rash	Targeted Therapy Re-initiation	Additional Therapies	Disease Status after Rash
1	IVFs	Dexamethasone 10 mg q6h inpatient then prednisone 1 mg/kg followed by extended taper outpatient	42 days prescribed (however remained on steroids for entire duration of targeted therapy)	39 days	Yes, D/T 44 days after rash onset On prednisone 10 mg daily	Temozolomide	Mixed response at 1 month, significantly worsened disease at 2 months
2	IVFs	Prednisone 150 mg daily inpatient followed by extended taper outpatient	53 days	37 days	Yes, D/T 39 days after rash onset On prednisone 10 mg daily	Ipilimumab Pembrolizumab CD40 agonist/ Pembrolizumab	Mixed response at 1 month, stable disease at 1 year, worsened disease at 18 months
3	IVFs	Methylprednisolone 50 mg daily inpatient followed by prednisone 60 mg daily with extended taper outpatient	17 days after first rash onset For duration of targeted therapy after rash recurrence	16 days	Yes, V/C (dose reduced) 24 days after rash onset Off prednisone Immediate fever and rash, held additional 13 days then restarted while on prednisone 30 mg daily	None	Mixed response at 1 month, improved disease at 4 months, worsened systemic disease at 8 months, worsened intracranial disease at 9 months
4	None	Prednisone 20 mg daily for 3 days, 10 mg daily for 3 days	6 days	12 days	Yes, V/C 13 days after rash onset Off prednisone Recurrence of rash after 1st dose, discontinued again Restarted 59 days after rash onset Immediate grade 4 allergic reaction to vemurafenib Tolerated D/T 166 days after initial rash onset when started with concurrent steroids	Temozolomide D/T Ipilimumab	Improved disease at 1 month, mixed response at 3 months (before temozolomide), worsened disease at 5 months (on temozolomide) improved response at 9 months (after initiation of D/T), worsened disease at 13 months
5	IVFs, Vasopressors	Methylprednisolone 70 mg BID inpatient followed by prednisone 60 mg daily with extended taper outpatient	54 days at time of readmission for rash recurrence when tapered to prednisone 5 mg daily, prolonged taper again thereafter	15 days	No	Temozolomide	Disease progression at 2 months (before temozolomide), worsened disease at 5 months
6	None	Prednisone 10 mg daily followed by extended taper	Ongoing (> 50 days)	6 days	Yes, V/C 7 days after rash onset On prednisone 10 mg daily	None	No disease progression

*IVFs* Intravenous fluids, *V* Vemurafenib, *C* Cobimetinib, *D* Dabrafenib, *T* Trametinib

(Table 2) Apart from supportive care, corticosteroids were an integral part of the management of the CAE. The dose and duration for steroid use were based on the severity of the initial presentation. Patients that had more severe clinical presentations were started on higher doses with a protracted taper (patient-2, 3 and 5) while as patients that had relatively milder clinical presentations and lower intensity rash (for example patient-4 and 6) were started on lower doses which were continued for a shorter period of time. After stopping the treatment and initiating supportive care, by week three resolution in the CAE was observed in a majority (patients 3–6), while patients-1 and 2 took approximately 6 weeks for the rash to resolve. ICB or TT was reinitiated in five patients within 1–8 weeks after resolution of the index CAE. This resulted in two patients (Patients 3 and 4) re-experiencing the CAE. Both of these patients were off prednisone at the time of therapy re-initiation, whereas none of the patients in our series who were restarted on TT with a steroid overlap (Patients 1, 2 and 6) had a rash recurrence. Patient 5 relapsed with a rash on day 54 after the index rash when she was tapered down to 5 mg prednisone. Following this, her dose for prednisone was increased again with a protracted taper. Patient 2 experienced controlled disease for more than 1 year after reinitiating TT without recurrence of adverse events. Patient 6 remains on TT, now greater than 6 months since reinitiating it. Despite the initial recurrence of rash in patient 3, he was able to be restarted on TT a second time, with steroid overlap, and to stay on this for greater than 9 months after that without additional CAEs. Patient 4 was switched to dabrafenib/trametinib (D/T) 166 days after the index CAE with gradual dose escalation, which she tolerated well despite rash recurrence when previously re-challenged with V/C. Subsequently, patient 4 had to be switched from D/T to ipilimumab due to disease progression and died within 1 month thereafter. Two of the six patients (patients 1 and 5) were noted to have disease progression on revaluation after 4–8 weeks of the CAE. In the entire cohort of 6 patients, two patients continue to be alive, receiving ongoing treatment (patient 6 on D/T and patient 2 on ICB).

## Discussion

The biological rationale of combining or sequencing ICB with TT in metastatic melanoma stems from a growing body of evidence supporting the favorable immune effects created by the oncogene-targeted therapies directed at the BRAF/MAPK pathway [4]. These beneficial effects that lead to potentiation of the immune effector function within the tumor microenvironment are mediated through various mechanisms, including effects on dendritic cell function, natural-killer-cell activation, increased HLA expression, upregulated expression of melanoma-derived antigens and T-cell homing to the tumor [5–7].

In this case series, we discuss cutaneous toxicity patterns in patients with B-RAF positive metastatic melanoma treated with sequential strategies involving ICB and TT in response to disease progression. Although very recently similar findings have been described by others in patients receiving TT after ICB [8], we provide a broader description of the unique histopathological characteristics and also attempt to address questions relating to management strategies as well as the feasibility of re-challenging patients with these agents in certain circumstances.

As shown in Table 1, five of the six patients received ICB before TT and tolerated anti-PD-1 therapy without any overt concerns of toxicity. This raises speculation of immune priming by ICB followed by immune boosting by the TT which may result in aberrant immunomodulation. Findings from a recently published report elucidate worse clinical presentations associated with cutaneous toxicities to vemurafenib in patients previously exposed to nivolumab [9], lending proof of concept to the hypothesis of durable activation of T-effector cells by prior anti-PD-1 therapy triggering hypersensitivity to TT. Interestingly, in our series where patients had prior ICB use, the occurrence the CAE usually manifested within the first two to 3 weeks from introduction of vemurafenib-based TT combinations. These findings are consistent with other reports of adverse cutaneous responses manifesting within a similar timeline in patients treated with vemurafenib after exposure to nivolumab [10, 11] and ipilimumab [12]. Most of these reported cases used single agent vemurafenib without concurrent MEK inhibition following ICB. This is in contrast to our series and a series by another group [8] where the B-RAF/MEK combination was used, albeit with similar findings. For now, mechanisms dictating augmented toxicity with vemurafenib containing combinations following ICB remain unclear, which has prompted us to modify our clinical practice by incorporating dabrafenib instead of vemurafenib for B-RAF inhibition when choosing TT combinations after disease progression on ICB.

Most of the patients had clinical features consistent with SIRS. In a subset of patients, these cutaneous adverse events were associated with a rise in makers of inflammation such as C-reactive protein (Table 1). It is notable to mention that in addition to other pro-inflammatory cytokines, one patient also had a high level of the soluble interleukin-2 receptor (IL-2R), a nonspecific marker of T-lymphocyte activation often used as one of the diagnostic criteria for hemophagocytic syndromes [13]. This could explain the constellation findings of SIRS with high a CRP, hypotension, and fever seen in our series. Similarly, in another report of two patients with TT after ICB, one patient had a similar presentation of rash with SIRS within 8 days of starting V/C after ICB [14]. Although findings attributed to potential macrophage activation syndrome with D/T after ICB have been reported in the literature [15], no accompanying cutaneous features were observed. Thus a deeper understanding of the role of altered cytokine physiology during these adverse events may facilitate in better management strategies.

Given that none of our patients had clinical or histological features concerning for Stevens - Johnson syndrome (SJS) and a majority were noted to have disease progression, an attempt to restart TT (patients 1–4 and patient-6) was made within 1–8 weeks after the rash. We observed that delayed re-exposure to the treatment after completing a steroid taper resulted in rash reoccurrence whereas re-exposure with steroid overlap was not associated with rash reoccurrence in our case series. This again is suggestive of a persisting immune activation despite drug discontinuation, a feature commonly attributed to the prolonged pharmacodynamic activity of ICB which perhaps can be ameliorated using steroids when opting to re-challenge these patients with TT. Also, of the five patients who were restarted on TT, four were able to stay on it with good effect for > 200 days after re-initiation. Thus the possibility of achieving clinically meaningful benefit on re-challenging these patients with TT needs strong consideration (Table 3).

**Table 3:** Practical Advice for Clinical Management

- Consult dermatology and biopsy right away, photographs recommended to help document rash.	
- Consider obtaining CRP as surrogate for IL-6 as patients may present with SIRS.	
- Consider re-initiation of therapy after rash (at lower dose, with steroid overlap), particularly if no signs of SJS, biopsy appears benign relative to clinical rash, and patient was having a good response to the therapy or does not have alternate therapy options.	
- Consider avoidance of other stimulating medications or known activating medications such as amoxicillin, amoxicillin-clavulanic acid, allopurinol	

Due to the clinical presentation and the pattern of the diffuse morbilliform rash, Drug Reaction with Eosinophilia and Systemic Symptoms (DRESS) syndrome was considered a leading differential in four of our six patients. Our patients lacked the severe transaminitis, eosinophilia, and extended latency periods commonly seen in DRESS syndrome, but did have features that would support a diagnosis of “possible” or “probable” cases by RegiSCAR criteria [16], including fevers, facial swelling, and duration of the rash. Interestingly, this atypical pattern of DRESS syndrome, lack of eosinophilia and shorter latency periods for grade-4 rash, has been described in other series of patients receiving TT following ICB [8]. Nevertheless, a high index of suspicion for SJS or toxic epidermal necrolysis (TEN) should be maintained in all patients presenting with rapid-onset, diffuse rashes after changes in drug therapy. Although two of our patients had mucositis, this was limited to the oral mucosa, and the remainder of the rash did not blister or desquamate which again did not support SJS. In cases such as these, a skin biopsy can be particularly helpful in predicting the clinical course.

Despite the extensive nature of the rash on clinical exam, the pathologic findings in the skin biopsies for our cases were relatively mild and primarily characterized by papillary dermal edema with a lympho-eosinophilic infiltrate (Fig. 3). This pattern of inflammation suggests a delayed hypersensitivity type reaction. None of the biopsies showed toxic epithelial changes or interface alterations suggestive of SJS/TEN, erythema multiforme-like drug reactions or vasculitis. In contrast, In contrast, the biopsy from patient-2 displayed distinctive features of sub-epidermal vesicle formation and positive immunofluorescence studies supportive of an immunobullous disease. Given that patient 2 was on TT for a long duration without any CAEs, we believe that the use of an antibiotic in patient-2 for possible pneumonia (Table 1) may have worked in conjunction with the TT (D/T) to produce the dramatic clinical picture along with the cutaneous findings. In general, the scarcity of the inflammatory cells and lack of toxic or necrotizing changes in the cutaneous vessels and epithelium were uniting histopatholic features in our skin specimens.

An important point to note is the timeline of onset and severity of the CAEs. The median time to onset of rash in our series was 14.5 days. Development of a rash within 5–14 days is typical for a morbilliform or simple drug eruption; in contrast, patients with a systemic drug hypersensitivity eruption often have a more delayed presentation 3–6 weeks after drug initiation. Our patients had features of systemic hypersensitivity but presented earlier than might be expected for typical drug-induced hypersensitivity or DRESS syndrome. This seems distinct from the rash that patients have TT without history of prior immunotherapy in which the duration of medication exposure prior to rash onset is longer with less severe systemic symptoms. This could support the hypothesis that exposure to prior immunotherapy led to durable immune alterations which would then augment a hypersensitivity drug reaction caused by TT, thus explaining the more rapid onset and the severity of symptoms, a mechanism proposed by others too [14]. For now more pre-clinical data to identify molecular mechanisms linking ICB to TT that could drive these CAEs is required to support these claims.

Several trials currently underway are aimed at elucidating the utility of using a two-pronged approach of combining TT and ICB as well as sequencing these agents [4, 17, 18]. A highly anticipated trial looking into the optimal sequencing of TT and ICB is the ECOG phase III study [19] estimated to have results reported by 2022. It is hopeful that the results of these trials will provide definitive information on the optimal strategy for sequencing of TT and ICB and thus could have significant implications on influencing treatment decisions in advanced B-RAF mutated melanoma in the near future.

In conclusion, our cases highlight the importance of maintaining a high index of suspicion for toxicities especially involving the skin when opting for strategies that involve sequencing of TT and ICB more so in the first two to 3 weeks of the switch. Findings from our cases show that these adverse clinical presentations with associated cutaneous findings tend to respond well to supportive measures and steroids. Also, depending on the disease status and the grading of the reactions, it may be reasonable to re-challenge select patients under strict supervision preferably with a steroid overlap even after rash resolution; keeping in mind that persisting immune activation from ICB may portend a high risk of rash reactivation despite several weeks of steroid use. Given the lack of prospective studies so far, most of our experiences with such agents administered either sequentially or in combination are guided by retrospective data similar to ours. Hence unraveling principles governing optimal sequencing or mechanisms contributing to potential toxicity patterns in patients receiving these treatments requires further understanding with prospective evidence.

## References

[1] Luke JJ, Flaherty KT, Ribas A, Long GV (2017). Targeted agents and immunotherapies: optimizing outcomes in melanoma. Nat Rev Clin Oncol.

[2] Eroglu Z, Ribas A (2016). Combination therapy with BRAF and MEK inhibitors for melanoma: latest evidence and place in therapy. Ther Adv Med Oncol.

[3] O’reilly A, Larkin J (2017). Checkpoint inhibitors in advanced melanoma: effect on the field of immunotherapy. Expert Rev Anticancer Ther.

[4] Atkins MB, Larkin J (2016). Immunotherapy Combined or Sequenced With Targeted Therapy in the Treatment of Solid Tumors: Current Perspectives. JNCI: J Natl Cancer Inst.

[5] Hu-Lieskovan S, Mok S, Moreno BH, Tsoi J, Faja LR, Goedert L (2015). Improved antitumor activity of immunotherapy with BRAF and MEK inhibitors in BRAF(V600E) melanoma. Sci Transl Med.

[6] Hu-Lieskovan S, Robert L, Homet Moreno B, Ribas A (2014). Combining targeted therapy with immunotherapy in BRAF-mutant melanoma: promise and challenges. J Clin Oncol.

[7] Cooper ZA, Juneja VR, Sage PT, Frederick DT, Piris A, Mitra D (2014). Response to BRAF inhibition in melanoma is enhanced when combined with immune checkpoint blockade. Cancer Immunol Res.

[8] Lamiaux M, Scalbert C, Lepesant P, Desmedt E, Templier C, Dziwniel V (2018). Severe skin toxicity with organ damage under the combination of targeted therapy following immunotherapy in metastatic melanoma. Melanoma Res.

[9] Imafuku K, Yoshino K, Ymaguchi K, Tsuboi S, Ohara K, Hata H (2017). Nivolumab therapy before vemurafenib administration induces a severe skin rash. J Eur Acad Dermatol Venereol.

[10] Satoshi T, Koji Y, Kei Y, Keisuke I, Kuniaki O (2017). Two cases of successful treatment for severe skin rash induced by vemurafenib following nivolumab therapy without cessation of vemurafenib. J Dermatol.

[11] Johnson DB, Wallender EK, Cohen DN, Likhari SS, Zwerner JP, Powers JG (2013). Severe cutaneous and neurologic toxicity in melanoma patients during vemurafenib administration following anti-PD-1 therapy. Cancer Immunol Res.

[12] Harding JJ, Pulitzer M, Chapman PB (2012). Vemurafenib sensitivity skin reaction after ipilimumab. N Engl J Med.

[13] Lin M, Park S, Hayden A, Giustini D, Trinkaus M, Pudek M (2017). Clinical utility of soluble interleukin-2 receptor in hemophagocytic syndromes: a systematic scoping review. Ann Hematol.

[14] Urosevic-Maiwald M, Mangana J, Dummer R (2017). Systemic inflammatory reaction syndrome during combined kinase inhibitor therapy following anti-PD-1 therapy for melanoma. Ann Oncol.

[15] Umemura H, Yamasaki O, Morizane S, Iwatsuki K (2017). Possible macrophage activation in melanoma patients receiving combined kinase inhibitor therapy following anti-PD-1 therapy: a cytokine profiling study of two cases. Ann Oncol.

[16] Kardaun SH, Sidoroff A, Valeyrie-Allanore L, Halevy S, Davidovici BB, Mockenhaupt M (2007). Variability in the clinical pattern of cutaneous side-effects of drugs with systemic symptoms: does a DRESS syndrome really exist?. Br J Dermatol.

[17] Ribas A, Hodi FS, Lawrence DP, Atkinson V, Starodub A, Carlino MS (2016). Pembrolizumab (pembro) in combination with dabrafenib (D) and trametinib (T) for BRAF-mutant advanced melanoma: Phase 1 KEYNOTE-022 study. J Clin Oncol..

[18] ClinicalTrials.gov. A Study of Atezolizumab Plus Cobimetinib and Vemurafenib Versus Placebo Plus Cobimetinib and Vemurafenib in Previously Untreated BRAFv600 Mutation-Positive Patients With Metastatic or Unresectable Locally Advanced Melanoma [updated July 10, 2018. Available from: https://clinicaltrials.gov/ct2/show/NCT02908672.

[19] ClinicalTrials.gov. Dabrafenib and Trametinib Followed by Ipilimumab and Nivolumab or Ipilimumab and Nivolumab Followed by Dabrafenib and Trametinib in Treating Patients With Stage III-IV BRAFV600 Melanoma [updated July 13, 2018. Available from: https://clinicaltrials.gov/ct2/show/NCT02224781.

